# Editorial: Beyond humans—Virus therapy for pathogens of animals and plants

**DOI:** 10.3389/fmicb.2022.1127901

**Published:** 2023-01-05

**Authors:** Eeva J. Vainio, Hany Anany, Paul Hyman

**Affiliations:** ^1^Forest Health and Biodiversity, Natural Resources Institute Finland (Luke), Helsinki, Finland; ^2^Guelph Research and Development Centre, Agriculture and Agri-Food Canada, Guelph, ON, Canada; ^3^Department of Food Science, University of Guelph, Guelph, ON, Canada; ^4^Department of Biology and Toxicology, Ashland University, Ashland, OH, United States

**Keywords:** virus, bacteriophage, mycophage, phage therapy, virotherapy, plant pathogens, animal pathogens, fungal pathogens

Phage therapy was originally developed by Felix d'Herelle and others to treat human bacterial diseases (Summers, 1999) and much of the history of phage therapy has focused on those same diseases (Chanishvili, 2012). But bacteria cause diseases in animals and in plants and there is no reason phages can't have applicability to those diseases. This idea was pursued by d'Herelle who tested the use of a *Salmonella gallinarum* phage to protect chickens during a fowl typhoid outbreak in 1919 (Sulekvelidze and Barrow, 2005). More recently, especially with the rise of antibiotic resistance in bacteria, phage therapy of human bacterial diseases has been widely written about with fewer publications on diseases of other species. This in spite of the fact that phage products for controlling several plant diseases have been marketed in the United States for almost 20 years (https://www.agriphage.com/) and products for decontaminating surfaces and foods are also being marketed for over a decade (Loc-Carrillo and Abedon, 2011). To address non-human phage therapy, we conceived of this research collection. We also chose to broaden the topic area beyond phage to include viruses of eukaryotic pathogens, protists and fungus. Thus, one can use the more general term virus therapy or virotherapy to describe this research. Broadly, we can identify three groups of pathogens that have been studied as potentially treatable by virotherapy: bacterial pathogens of animals; bacterial pathogens of plants; and non-bacterial pathogens of animals or plants.

## Bacterial pathogens of animals

As noted above, the first attempt to treat animals was tried by d'Herelle after he first identified bacteriophage but before their identity as viruses was known. W.W.C. Topley, an early phage researcher noted in his 1925 public lecture on “the bacteriophage principle” that “it is a conceivable hypothesis that the administration of a lytic filtrate, active against bacterium A, to animal B, in whose tissues bacterium A is multiplying with disastrous results, will terminate the conflict in B's favor” (Topley, 1925). In the 1920s and 1930s, many researchers attempted to treat animals with bacterial infections, mainly in laboratory studies (Sulekvelidze and Barrow, 2005). Results were mixed sometimes due to not understanding the need to use phages capability of lysing the target bacteria.

Phage therapy improved as the viral nature of bacteriophages was understood but there was a decreased interest in phage therapy in much of the world as antibiotics became widely available. Phage therapy continued to be used in some countries, especially Georgia and Poland (Sulekvelidze and Kutter, 2005). But in recent decades, the rise of antibiotic-resistant bacteria has prompted renewed interest in phage therapy of human and animal pathogens. Animal diseases studied for treatment include agriculturally important species such as poultry (Abbas et al., 2022) and cattle (Ngassam-Tchamba et al., 2020) but also aquaculture species (Kalatzis et al., 2018).

## Bacterial pathogens of plants

Early work on phage therapy of plant diseases also began in the 1920s. Moore reported in 1926 on the detection and testing of an “invisible agent” to treat Wildfire disease of tobacco, citing d'Herelle's work as a basis of the study (Moore, 1926). Subsequent studies were published but lagged behind animal studies (Sulekvelidze and Barrow, 2005). These focused on various species including corn and peaches. In the last 20 years, there has been an increased examination of bacteriophages as a “natural” alternative to antibiotics. This has led to the longest commercialization of phages as biocontrol agents in the United States. In 2005, Omnilytics Inc. (Sandy, Utah, USA; https://www.omnilytics.com/) began selling customized bacteriophage mixtures for use against plant pathogens. Initially the target diseases were tomato and pepper bacterial spot diseases caused by *Xanthomonas* and *Pseudomonas* species but they now provide phages against the pathogens causing tomato bacterial canker (*Clavibacter michiganensis*); apple and pear fire blight (*Erwinia amylovora*); and citrus canker (*Xanthomonas citri*). Much of this product development built, of course, on experimental work published previously (Gill et al., 2003; Obradovic et al., 2004). In Europe, biocontrol products are assessed by a similar regulatory pathway as synthetic pesticides. No phages have been so far registered in Europe, but member states can give provisional authorization under specific circumstances (Wagemans et al., 2022). Currently there are commercially available phage products in Europe: a phage cocktail against *E. amylovora* sold in Hungary, and APS Biocontrol (Dundee, UK, https://www.apsbiocontrol.com/) sells a product against *Pectobacterium* soft rot of potato.

## Virus therapy of non-bacterial pathogens

Virus therapy/virotherapy follows the same approach as phage therapy, using viruses as treatments for diseases caused by the virus host. This approach has been discussed for protozoan pathogens of animals (and humans) but, to date, has not been implemented in any significant trials (Barrow et al., 2020). Similarly, currently known viruses of important oomycete pathogens, such as the potato blight pathogen *Phytophthora infestans*, have not proved suitable for biocontrol applications and may actually increase oomycete fitness (Hillman and Milgroom, 2021). A more developed application is the treatment of fungal diseases of plants with fungal viruses (mycoviruses) specifically the treatment of chestnut blight which is caused by a fungus, *Cryphonectria parasitica*. However, these viruses don't kill the fungus but rather slow down its growth rate, reducing its ability to attack chestnut trees (Myers and James, 2022; Wagemans et al., 2022). These viruses are described as being “hypovirulent” and have been widely employed in treating chestnut trees by infecting them with hypovirulent virus-infected fungus to spread the virus. This approach is being studied for other fungal pathogens as well. Scientists have already identified viruses that delimit the growth and/or virulence of other plant pathogens (García-Pedrajas et al., 2019), but their development into commercial products is a slow process (Wagemans et al., 2022).

In summary, while most discussions of viruses as biocontrol agents focus on phage therapy of human diseases, there are a great many other applications of this approach. Some, like the Agriphage products for plant diseases and the hypovirulent mycoviruses of chestnut blight fungus, are already in widespread use. Thus, the future seems bright for these broader applications beyond human disease.

## Author contributions

PH and EV prepared the editorial. HA edited the editorial. All authors contributed to the article and approved the submitted version.
